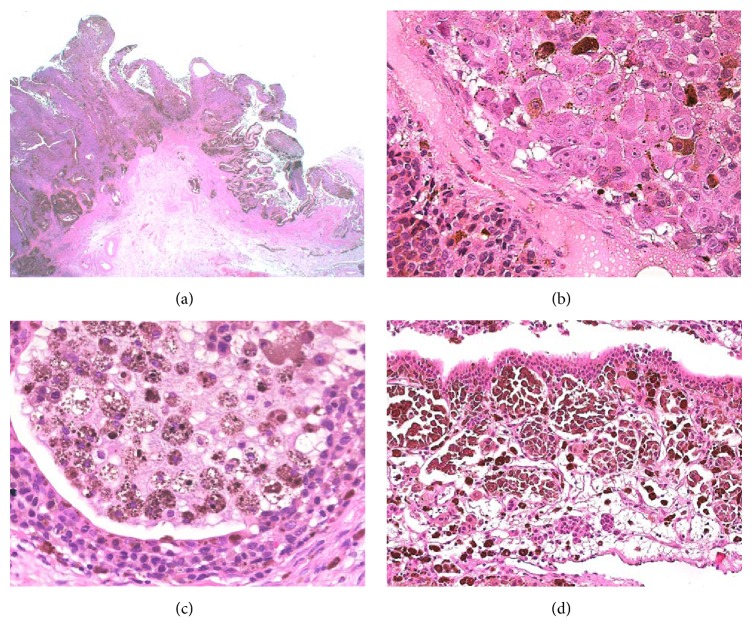# Clinicohistopathological Characteristics of Malignant Melanoma in the Gall Bladder: A Case Report and Review of the Literature

**DOI:** 10.1155/2018/6471923

**Published:** 2018-03-20

**Authors:** Schmidt Adrian, Caspar Clemens, Schmidt-Weiss Elisabeth, Stadlmann Sylvia

**Affiliations:** ^1^Department of Internal Medicine, Division of Medical Oncology and Hematology, Triemli Hospital, Birmensdorferstrasse 497, 8063 Zurich, Switzerland; ^2^Department of Internal Medicine, Division of Medical Oncology and Hematology, Cantonal Hospital Baden, Im Ergel 1, 5404 Baden, Switzerland; ^3^Centre for Hematology and Oncology, Clinic of Medical Oncology, University Hospital Zurich, Rämistrasse 100, 8091 Zürich, Switzerland; ^4^Division of Pathology, Cantonal Hospital Baden, Im Ergel 1, 5404 Baden, Switzerland

## Abstract

**Objective:**

Primary gall bladder melanoma is a rare and controversial entity. So far, only 36 cases are documented in the literature. Metastatic melanoma targeting the gall bladder, however, has been reported to occur in about 15–20% of melanoma patients and is much more common.

**Methods:**

Based on the case of a 58-year-old woman presenting with multiple melanoma nodules in the gall bladder, we searched in the available literature in PubMed for articles describing a “primary melanoma of the gallbladder” regardless of language used.

**Results:**

We detected 33 papers that described 36 cases of primary gall bladder melanoma between 1907 and 2017. From different criteria distinguishing primary and secondary gall bladder melanoma, generally, the following were accepted: (1) exclusion of previous primary melanoma, (2) absence of synchronous involvement of other sites, (3) unicity of the lesion, (4) polypoid or papillary shape of the lesion, and (5) presence of junctional melanocitary component. Review of the 36 published cases revealed that only about one-fourth of them fulfilled all the five criteria.

**Conclusion:**

Primary gall bladder melanoma is even rarer than described in the literature, and the question whether this entity really exists remains open.

## 1. Introduction

The existence of a primary melanoma of the gall bladder is controversial, and only about 40 cases are described in the literature so far. Based on an own case with diagnosis of a melanoma in the gall bladder, we were wondering which criteria should be fulfilled for this diagnosis, how they are able to separate this entity from metastatic melanoma, and how these criteria would match with the formerly reported cases as well as with the history of our own patient.

## 2. Case Report

**Figure 1:**
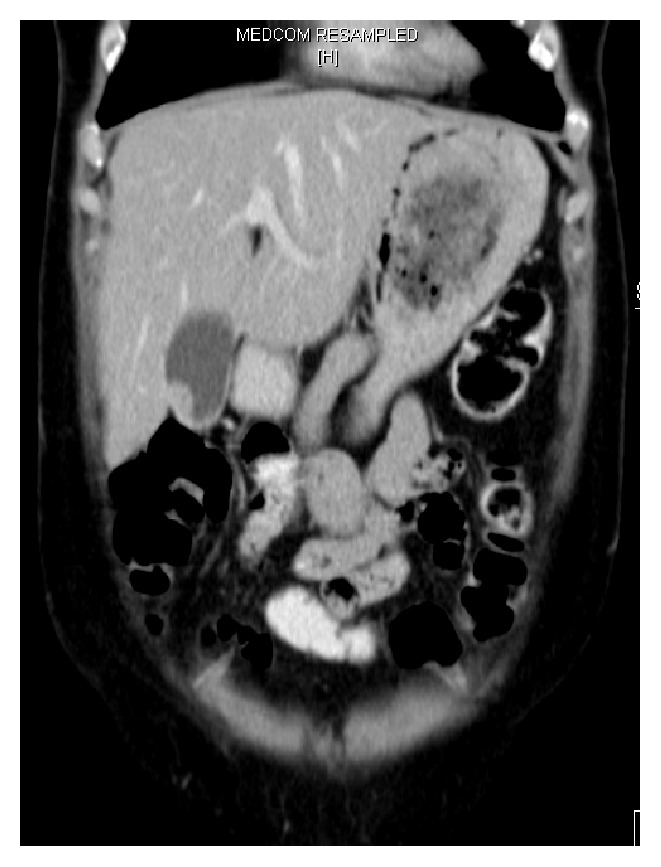


A 58-year-old Caucasian woman presented at our institution with immobilizing back pain, known for 4 years but exacerbating for 3 months. The past history included smoking (about 15 pack-years), appendectomy and tonsillectomy as a child, and extensive endometriosis which required surgical excision of sigmoid colon and hysterectomy sixteen years earlier. Conventional X-rays of the thoracic and lumbar spine revealed degenerative alterations only. However, magnetic resonance imaging (MRI) demonstrated multiple lesions in all vertebral bodies with a pathologic fracture in the 8th thoracic vertebral body. Blood count was completely normal. Suspecting bone metastases of a yet unknown primary tumour, a thoracoabdominal CT scan was performed, revealing disseminated small nodules in both lungs (max. 1.0 × 0.5 cm in diameter) and small polypoid intraluminal lesions in the gall bladder infiltrating the liver (Figure 1), the latter showing additionally some very small lesions measuring only a few millimeters in diameter. An ultrasound confirmed a solid tumour mass in the fundus of the gall bladder, highly suspicious for primary gall bladder cancer. Upper endoscopy and lower endoscopy were inconspicuous. External biopsies of a suspected pulmonary lesion and the thickened conglomerate of gall bladder wall towards liver were both not diagnostic. Repeating one of these interventions seemed not promising. Palliative radiotherapy (5 × 4 Gy) of the thoracic spine was performed. Unfortunately, severe mood depression and panic attacks occurred, delaying further diagnostic and therapeutic interventions. Six weeks later, a repeated CT scan revealed progression of the bone lesions and significant thickening of the gall bladder wall. MRI did not detect brain metastases. For further diagnosis, we discussed with the patient pulmonary wedge resection, vertebral biopsy, or cholecystectomy. With the two unsuccessful attempts in mind, she chose the latter procedure, suggesting the highest probability to finally reach a diagnosis. This intervention was then performed without complications and provided the following findings.

Macroscopically, the gall bladder specimen was 9 cm long and 3 cm in diameter. In the lumen, a friable broad based papillary nodule in the fundus (5 cm maximum diameter) and two polypoid satellite nodules in the neck (1 cm maximum diameter) were present. In the cystic duct, a small gallstone was identified. Histopathologically, surprisingly, the nodules corresponded to a malignant melanoma. The tumour was almost completely confined to the mucosa (Figure 2(a)) with only focal invasion of the muscularis propria and subserosa. It was composed of pigmented polygonal cells with vesicular nuclei and prominent eosinophilic nucleoli (Figure 2(b)). Numerous mitotic figures could be encountered. The lining of the villi of the gall bladder mucosa consisted of tall columnar cells with in-between lying pigmented neoplastic cells and macrophages (Figure 2(c)). Junctional activity, meaning presence of microscopic aggregates of melanoma cells at the junction of epithelium and lamina propria, was present (Figure 2(d)). Tumour cells stained positively for S100, HMB45, Melan A, and CD117 by immunohistochemistry and melanin pigment with Fontana-Masson stain. Neither PDGFRA nor kit mutations were detected.

As neither BRAF inhibitors nor checkpoint inhibitors were yet available in clinical practice, a palliative chemotherapy with temozolomide (200 mg/m^2^ daily for 5 days) and monthly infusions of zoledronic acid were installed. Temozolomide was preferred to the former standard of care Dacarbazine, as equal efficacy was demonstrated [1] and the oral application convened to the patient.

Unfortunately, the patient developed severe myelosuppression CTCAE grade 4, requiring several transfusions of erythrocytes and platelets, as well as the application of G-CSF. Another CT scan performed after first chemotherapy cycle showed progression of the lung and liver metastases, newly developed spleen metastases, and pathologic fractures of the lumbar spine on levels 3 and 5. Because of the severe side effects without rendering any effect on tumour progression, the patient refused another series of palliative radiotherapy as well as another attempt of systemic therapy and decided to look for alternative treatment regimens. Two months later, she presented again with exacerbating bone pain, severe anemia (hemoglobin: 5.8 g/dl), and thrombocytopenia (36 G/l) as a result of progressive bone marrow infiltration. After installation of an analgesic therapy, the patient was transferred to a hospice institution, where she died a few days later.

## 3. Review of the Literature

Malignant melanoma in the gall bladder is rare. It usually represents a metastasis from a primary skin or mucosal melanoma and has been reported to occur in 15–20% of melanoma patients [2, 3]. Primary melanoma of the gall bladder is even much rarer with only 36 cases documented in the literature so far [4–36]. 15 (41.7%) of the reported patients were men and 21 (58.3%) were women. This corresponds to a male to female ratio of 0.71; range of age was 25 to 75 years at the time of diagnosis (median: 51.5 years). In none of the reported cases, a primary skin or mucosal melanoma could be identified. 27 of 31 (87.1%) evaluable patients initially presented with abdominal pain. 21 of 30 (70.0%) patients had symptoms of acute cholecystitis, demonstrating calculi in only 4 of 33 (12.1%) patients. Isolated tumours in the gall bladder without synchronous involvement of other organs at the time of diagnosis occurred in 22 of 35 (62.8%) patients. Unicity of the intraluminal gall bladder tumour was present in 25 of 35 (71.4%) patients. The median tumour size was 2.35 cm (range: 0.9–10 cm). 9 of 17 (52.9%) tumours were located in the fundus, 8 of 18 (44.4%) in the corpus, and/or 6 of 18 (33.3%) in the neck of the gall bladder. 12 of 25 (48.0%) tumours were pedunculated and 13 (52.0%) were broad based. 11 of 23 (47.8%) tumours infiltrated the muscularis propria and 3 of 18 (16.7%) and 4 of 21 (19.0%) cases additionally showed subserosal and/or serosal involvement. In 3 of 31 (9.7%) patients, malignant melanoma of the gall bladder resulted in perforation and acute peritonitis. Histomorphologically, the vast majority of melanoma cells exhibited epithelioid morphology (84.0%); 33.3% of cases had additional or exclusive sarcomatoid growth pattern. 29 of the 36 (80.6%) patients had cholecystectomy. 6 of 34 (17.6%) patients additionally received chemotherapy and 3 (8.8%) received radiotherapy. 11 of 33 (33.3%) patients developed lymph node metastases and 22 of 36 (61.1%) developed hematogenous metastases. 18 of 21 (85.7%) patients died of disease within 0.2 to 168 months after primary diagnosis (median: 9.0 months).

## 4. Discussion

Whether primary malignant melanoma of the gall bladder exists is controversial [32, 37]. Objective criteria for distinguishing primary melanoma of the gall bladder from metastatic melanoma include (1) exclusion of previous primary melanoma, (2) absence of synchronous involvement of other sites, (3) unicity of the lesion, (4) polypoid or papillary shape of the lesion, and (5) presence of junctional melanocitary component [38]. The presence of the latter has been described to be the most important diagnostic clue for primary gall bladder melanoma. However, in some reports of clearly melanoma metastases, histopathologically, a junctional activity was also detectable. Of the reported cases in the literature, only 8 of 29 meticulously documented patients (27.6%) fulfilled all of these 5 criteria. In our patient, junctional change at the tumour margin and a polypoid shape of the lesion could be clearly demonstrated. However, the presence of multiple tumour nodules in the gall bladder and synchronous involvement of other organs at the time of diagnosis favour a metastatic process. Reviewing the past history of our patient in fact revealed excision of a thoracic dysplastic nevus in an external hospital thirteen years earlier. Regarding clinicohistopathological findings, primary and secondary gall bladder melanomas share a remarkable similarity [18]. Based on our own and the reported data, a diagnosis of gall bladder melanoma should be favoured if (a) the patient has recurrent abdominal pain with symptoms of acute cholecystitis, (b) there is no evidence of cholecystolithiasis (which distinguishes it from gall bladder cancer), and (c) MRI or ultrasound reveals a polypoid intraluminal tumour confined to the gall bladder mucosa. The number of suspected primary gall bladder melanomas that withstand an accurate verification is very small, and it remains questionable if this entity really exists. Independently, surgical excision of suspected isolated gall bladder melanoma is mandatory.

## Figures and Tables

**Figure 2:**